# Aortic Unicuspid Valve Surgery in a Young Patient: A Case Report

**DOI:** 10.7759/cureus.75305

**Published:** 2024-12-08

**Authors:** Tomohiro Nakajima, Tsuyoshi Shibata, Yutaka Iba, Masayuki Akatsuka, Nobuyoshi Kawaharada

**Affiliations:** 1 Cardiovascular Surgery, Sapporo Medical University, Sapporo, JPN; 2 Department of Intensive Care Medicine, Sapporo Medical University, Sapporo, JPN

**Keywords:** aortic valve replacement, aortic valve stenosis, cardiac arrest, unicuspid valve, young

## Abstract

The patient was a 33-year-old male. He was noted to have a systolic murmur in the aortic valve region during childhood and underwent balloon valvuloplasty at a pediatric clinic. However, he was not followed up thereafter. Recently, he began experiencing exertional dyspnea and presented to our cardiology department. Detailed examinations, including echocardiography, revealed moderate aortic regurgitation and stenosis, and a unicuspid aortic valve was suspected morphologically. After discussing the findings with the patient, he opted for surgical treatment.

The operation was performed under general anesthesia via a median sternotomy. Intraoperative inspection of the aortic valve confirmed a unicuspid morphology, consistent with the preoperative findings. The unicuspid valve was excised and replaced with a mechanical valve (St. Jude Medical (SJM) 25 mm). The surgery was completed without complications, and the postoperative course was uneventful. The patient was discharged home on postoperative day 10.

Five years postoperatively, the patient remains asymptomatic and in good condition. Given the rarity of unicuspid aortic valve cases, this report is of significant clinical value.

## Introduction

The unicuspid aortic valve (UAV) is an extremely rare congenital anomaly, with a prevalence of approximately 0.02% in the general adult population [1]. This condition accounts for 4-6% of aortic valve surgeries performed for non-rheumatic aortic stenosis or regurgitation [2]. UAV typically manifests in two forms: unicommissural and acommissural, with the former being more common in adults. These anatomical variations result in severe valve dysfunction, often necessitating surgical intervention during early adulthood [3].

Unlike the more common bicuspid aortic valve, UAV is frequently underdiagnosed due to challenges in echocardiographic visualization. However, advancements in imaging techniques, including three-dimensional echocardiography and transesophageal approaches, have improved diagnostic accuracy. Despite these developments, UAV’s clinical course is marked by rapid deterioration once symptoms arise, particularly exertional dyspnea and syncope [4].

This report details a case of a 33-year-old male with a UAV, emphasizing the clinical presentation, diagnostic challenges, and surgical management. The case contributes to the growing understanding of this rare anomaly and its optimal treatment strategies.

## Case presentation

The patient was a 33-year-old male. During childhood, a systolic murmur in the aortic valve region was noted, and balloon valvuloplasty was performed at a pediatric clinic. However, no follow-up care was conducted afterward. After turning 30, he gradually developed exertional dyspnea and presented to our cardiology department at the age of 33.

**Figure 1:**
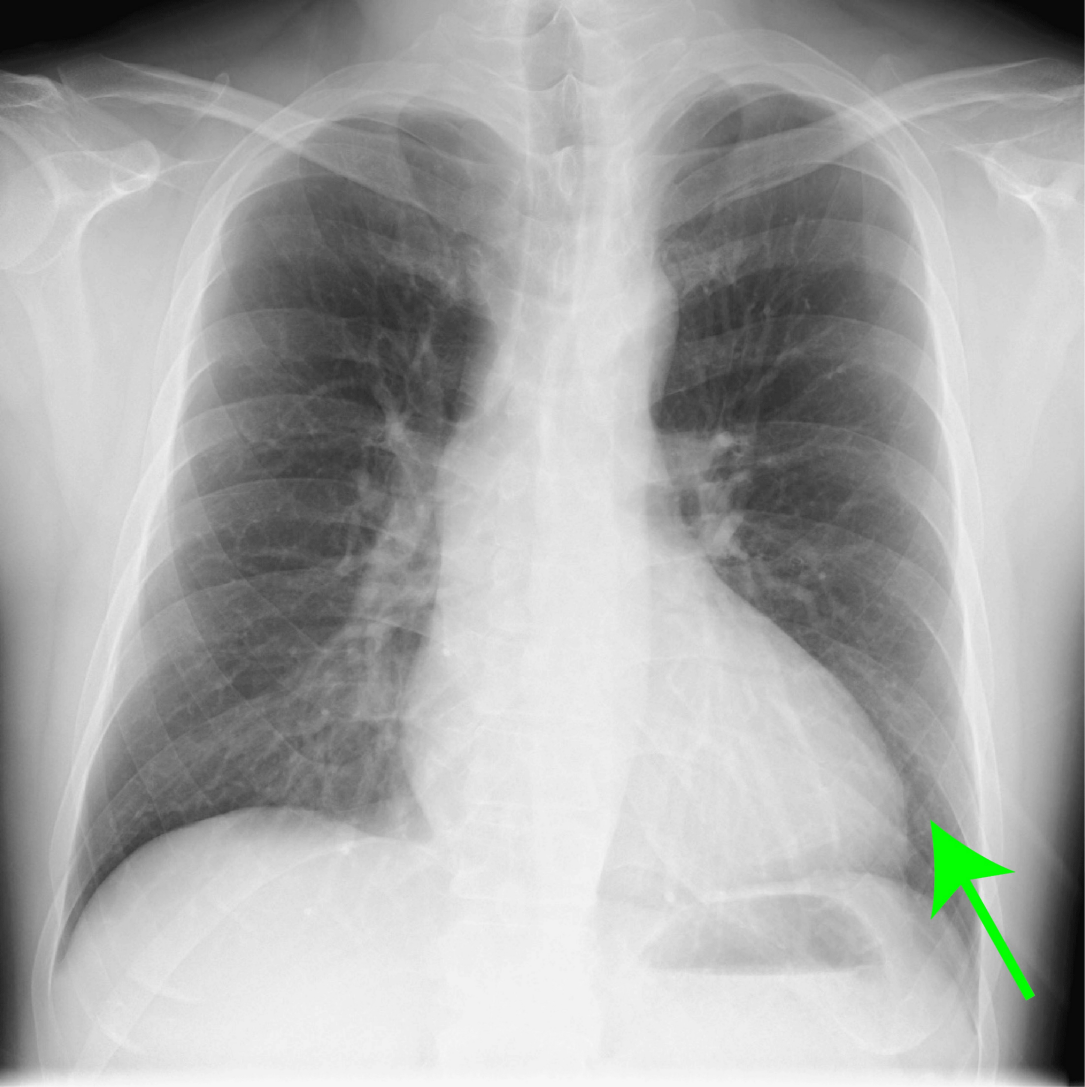
Preoperative chest radiograph. The cardiothoracic ratio was 0.50 and there was no pulmonary congestion (green arrow).

**Figure 2:**
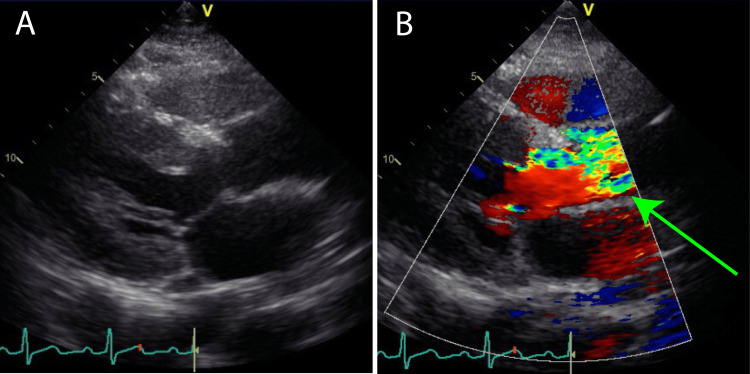
Preoperative echocardiography. (A) Long-axis image of the left sternal border. (B) The mosaic flow was observed at the aortic valve (green arrow).

**Figure 3:**
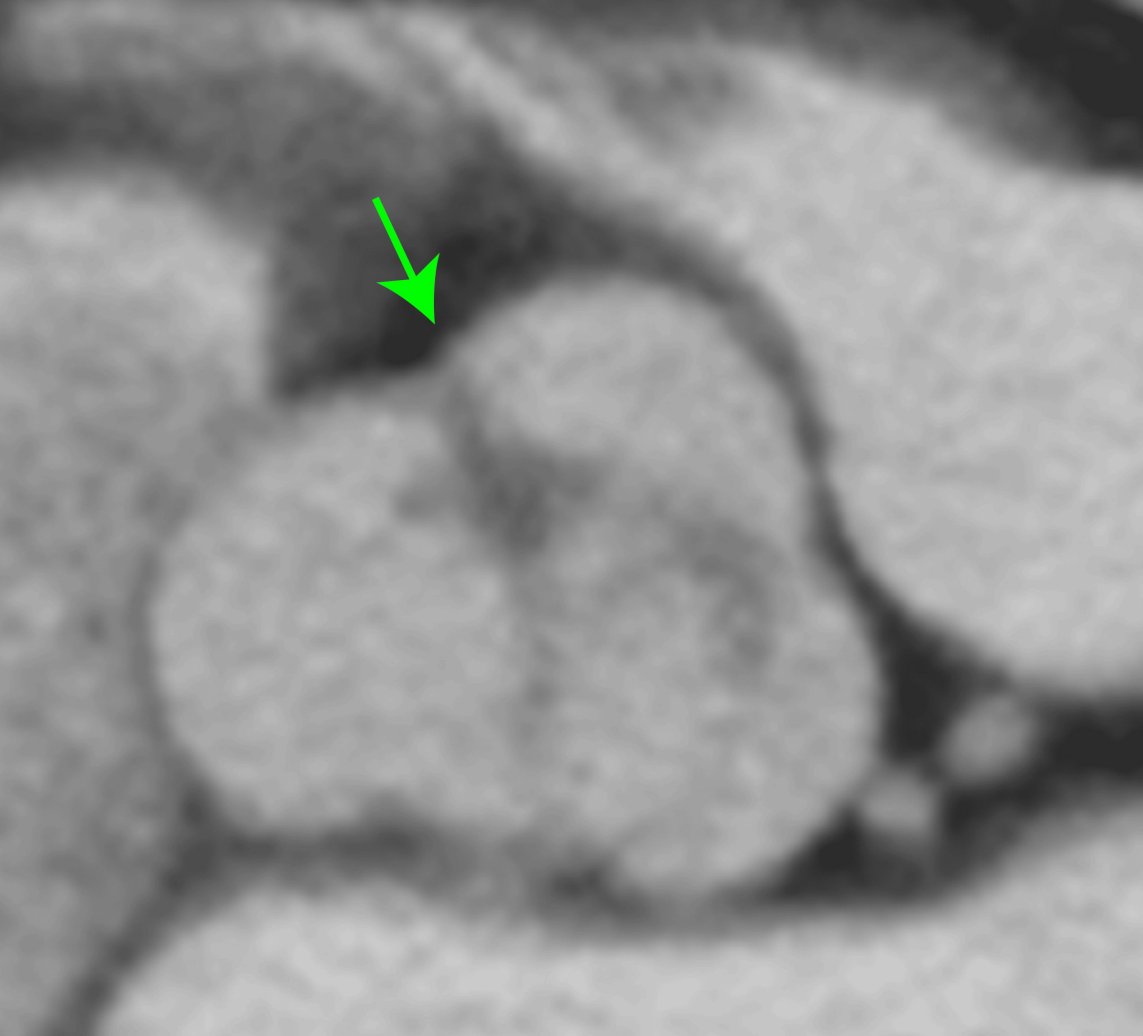
Preoperative enhanced computed tomography. Aortic valve view. The number of aortic valve leaflets was unknown (green arrow).

A chest X-ray showed no evidence of cardiomegaly or pulmonary congestion (Figure 1). Transthoracic echocardiography revealed moderate aortic regurgitation and stenosis, with morphological findings suggestive of a UAV (Figure 2). Preoperative contrast-enhanced computed tomography (CT) failed to clearly identify the number of valve cusps (Figure 3). After discussions with the patient, surgical intervention was planned.

The surgery was performed under general anesthesia through a median sternotomy. Cardiopulmonary bypass was established with arterial cannulation in the ascending aorta and venous cannulation in the superior and inferior vena cava. After aortic cross-clamping, cardioplegia was delivered selectively to the coronary arteries to achieve cardiac arrest, which was maintained with retrograde cardioplegia every 25 minutes.

**Figure 4:**
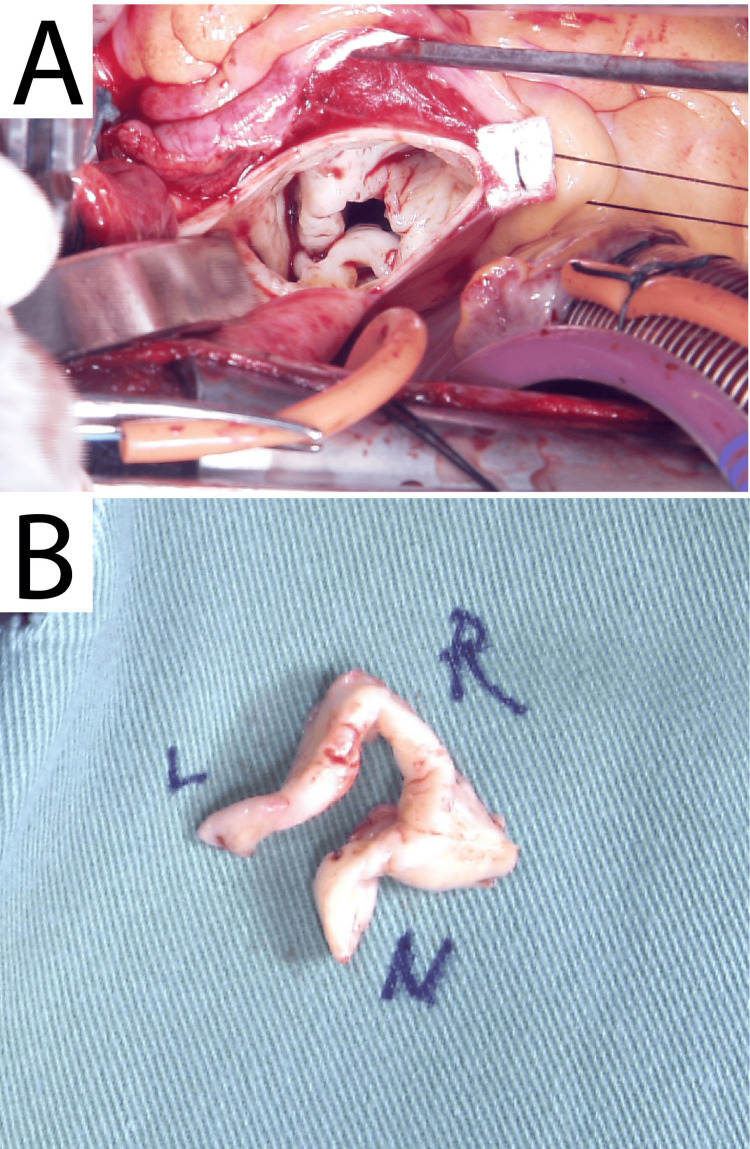
Intraoperative images. (A) Aortic valve before resection. All valves were fused, with a diagnosis of a unicuspid valve. (B) After resection.

A transverse incision in the ascending aorta exposed the aortic valve, confirming a unicuspid morphology as suspected preoperatively (Figure 4). The valve was excised, and a 25-mm St. Jude Medical (SJM) mechanical valve (St. Jude Medical, Inc., St. Paul, MN, USA) was implanted.

The procedure was completed without complications, and the postoperative course was uneventful. The patient was discharged home on postoperative day 10. Five years postoperatively, at the age of 38, he remains asymptomatic with no notable issues.

## Discussion

The UAV is an exceedingly rare congenital anomaly, with a prevalence of approximately 0.02% in the general population. It predominantly affects males and often presents earlier in life compared to bicuspid or tricuspid aortic valves due to the rapid progression of valve dysfunction. The rarity of UAV contributes to the limited understanding of its natural history and clinical outcomes [5].

The genetic underpinnings of UAV remain largely unknown. Unlike the bicuspid aortic valve, which has a well-established association with familial inheritance patterns, UAV appears sporadic in most cases. However, some studies have suggested potential links to specific genetic mutations involved in valve morphogenesis, warranting further research into its molecular basis.

Surgical intervention is the mainstay of treatment for symptomatic UAV, as medical management cannot reverse the structural abnormalities. Valve replacement, using either mechanical or bioprosthetic valves, is typically required due to the valve’s severe dysfunction. In this case, the choice of a St. Jude Medical mechanical prosthesis ensured long-term durability, reflecting the patient’s young age and the need to minimize reintervention risk [6]. Recently, transcatheter aortic valve implantation for the aortic unicuspid valve has been reported [7].

UAV is often associated with other congenital anomalies, including ascending aortic dilation, coarctation of the aorta, and left ventricular outflow tract obstruction. However, in this case, no significant extracardiac or associated structural abnormalities were identified, emphasizing the variability in clinical presentations of UAV [8].

The primary challenge in this case was the difficulty in preoperative diagnosis. Despite advancements in imaging modalities, including echocardiography and CT, identifying the unicuspid morphology preoperatively remains complex. Additionally, the lack of regular follow-up after childhood intervention highlights the importance of long-term surveillance in congenital valve abnormalities to prevent delayed presentations of severe symptoms.

## Conclusions

UAV is a rare congenital anomaly that often presents with severe valve dysfunction requiring surgical intervention. This case highlights the diagnostic challenges associated with UAV, even with advanced imaging modalities, and underscores the importance of long-term follow-up in congenital valve diseases. Successful surgical management with a mechanical prosthesis resulted in excellent postoperative outcomes, demonstrating the effectiveness of timely intervention. Further research into the epidemiology, genetics, and associated anomalies of UAV is crucial to improve early diagnosis and optimize management strategies.
